# Comparison of the Arterial PaCO _2_ Values and ETCO _2_ Values Measured with Sidestream Capnography in Patients with a Prediagnosis of COPD Exacerbation

**DOI:** 10.1055/s-0043-1771179

**Published:** 2023-07-13

**Authors:** Gökhan İşat, Tuba Cimilli Öztürk, Özge Ecmel Onur, Serdar Özdemir, Ebru Ünal Akoğlu, Fatma Tokgöz Akyıl, Hacer Kuzu Okur

**Affiliations:** 1 Department of Emergency Medicine, University of Health Sciences Umraniye Training and Research Hospital, Istanbul, Turkey; 2 Department of Emergency Medicine, University of Health Sciences Fatih Sultan Mehmet Training and Research Hospital, Istanbul, Turkey; 3 Department of Emergency Medicine, Marmara University, Istanbul Turkey; 4 Yedikule Center for Chest Diseases and Thoracic Surgery, Istanbul, Turkey; 5 Department of Pulmonary Medicine, Altunizade Acibadem Hospital, Istanbul, Turkey

**Keywords:** chronic obstructive pulmonary disease, capnography, blood gas analyses, carbon dioxide

## Abstract

**Background** Aim of this study is to investigate whether end-tidal carbon dioxide (ETCO _2_ ) values can be used instead of partial pressure of carbon dioxide (PaCO _2_ ) values in guiding treatment, and determining treatment benefits in patients that received a pre-diagnosis of chronic obstructive pulmonary disease (COPD) exacerbation at the emergency department.

**Methods** This observational prospective study was conducted with patients who presented to the emergency department with the complaint of shortness of breath and were diagnosed with COPD exacerbation. ETCO _2_ was measured with the sidestream method during blood gas analysis in patients with indications for this analysis. Measurements were repeated at hour 1 after treatment.

**Results** The study included a total of 121 cases. There was a positive correlation between the PaCO _2_ and ETCO _2_ values measured before and after treatment ( *r* = 0.736, *p* < 0.01 and *r* = 0.883, *p* < 0.01, respectively). High ETCO _2_ values were accompanied by high PaCO _2_ values. When the measurements before and after treatment were evaluated using the Bland–Altman method, most of the result were within the limits of agreement (−4.9 and +31.4/− 2.6 and +9.4), with mean differences being calculated as 13.2 and 8.4, respectively.

**Conclusions** Although ETCO _2_ and PaCO _2_ were statistically consistent according to the results of our study, due to the high averages of differences between these two parameters, the ETCO _2_ value has limited clinical use in COPD cases compared to PaCO _2_ . However, high ETCO _2_ values may indicate that noninvasive mechanical ventilation should be included in the treatment of COPD cases without waiting for the results of blood gas analysis, and they can also be when needed for inpatient treatment.

## Introduction

According to the estimates of the World Health Organization, there are approximately 65 million moderately severe cases of chronic obstructive pulmonary disease (COPD) in the world. Although the poor recognition of COPD and its diagnosis at advanced disease stage continue to affect epidemiological information, it is one of the leading causes of morbidity and mortality worldwide. 1 The economic burden of COPD is estimated to be 48.4 billion euros in the European Union. 2 In the United States of America, the direct economic burden is estimated at 32 billion dollars, and there is an additional 20 billion cost indirectly added to this burden. The severity of COPD is directly proportional to the cost of care, which is, in most cases, associated with the exacerbation of the disease. 3

Information about the metabolic and respiratory status of patients with COPD can be obtained by examining arterial blood gas parameters, including pH, partial pressure of oxygen (PaO _2_ ), partial pressure of carbon dioxide (PaCO _2_ ), base deficit, serum bicarbonate (HCO _3_ ), and oxygen saturation. 4 However, arterial blood gas collection is a painful procedure, and the frequency of complications increased in the presence of repeated interventions, which has led researchers to investigate noninvasive methods to determine the PaO _2_ and PaCO _2_ values that are considered to be the two cornerstones of treatment. Capnography, one of the methods developed for this purpose, measures the partial pressure of carbon dioxide from the airway during respiration. 5 With this noninvasive method, instant information can be obtained ventilation of the patient. Capnography has been used for patient follow-up in intensive care units for many years. 4 5 6

In this study, we aimed to investigate whether end-tidal carbon dioxide (ETCO _2_ ) values could be used instead of PaCO _2_ values in guiding treatment and determining treatment benefits in patients that received a diagnosis of COPD exacerbation at the emergency department.

## Material and Method

### Study Design and Population

This study was conducted prospectively and observationally at Emergency Department of Fatih Sultan Mehmet Training and Research Hospital and Emergency Department of Süreyyapaşa Chest Diseases and Chest Surgery Training and Research Hospital. The study group consisted of adult patients with a previous diagnosis of COPD, who presented to the emergency department of the two hospitals from April 1, 2015, through August 31, 2015, with the complaint of shortness of breath and received a diagnosis of COPD exacerbation. As defined in Global Initiative for Chronic Obstructive Lung Disease (GOLD), COPD exacerbation was accepted as an acute event characterized by the worsening of the patient's respiratory symptoms beyond the daily observed normal variability, resulting in changes in medication. In patients, the presence of at least one of the symptoms of worsening dyspnea, increased amount of sputum, and increased sputum purulence was sought. 7 Exclusion criteria included (i) no indication for arterial blood gas collection to make clinical diagnosis and management; (ii) patients whose first place of reference is not the emergency department (referrals) where the study was conducted; (iii) COPD exacerbation not confirmed by a pulmonologist; (iv) concurrently with one or more of the diagnoses of pulmonary edema, pulmonary embolism, and pneumonia; (v) mechanically ventilated patients; and (vi) patients who did not give their consent to participate in the study.

### Data Collection

Vital signs and anamnesis were taken. Following physical examinations, the blood gas analysis was performed in patients with relevant indications after obtaining their consent for this procedure. At the time when the clinician was taking the first blood gas samples, the first ETCO _2_ values of the patients were measured with the Masimo ISA capnograph using the sidestream method. After five to six spontaneous respirations, three or four simultaneous capnograms were observed on the monitor, and the ETCO _2_ value measured by the capnograph was recorded. Since the study was conducted observationally, the treatments applied to the patients were recorded without any interference. The control blood gas samples were taken by the clinician at the first hour after the treatment was completed, and the second ETCO _2_ measurements were undertaken. The blood gas samples taken from the patients were examined in the emergency laboratories of the two hospitals. The samples were taken with a blood gas injector containing 80 IU of electrolyte balanced heparin and examined in the ABL 700 blood gas analyzer.

### Statistical Analysis

For the statistical analyses of the data obtained from the study, IBM SPSS Statistics v. 22 was used. The conformity of the parameters to the normal distribution was evaluated with the Shapiro–Wilk test. In addition to descriptive statistical methods (mean, standard deviation, and frequency), the paired-samples *t* -test was used in the pre- and post-treatment comparisons of quantitative parameters in the presence of a normal distribution. The Mann–Whitney U test was conducted to compare the non-normally distributed parameters between the treatment groups. The Pearson correlation analysis was used to examine the relationships between PaCO _2_ and ETCO _2_ parameters, which conformed to the normal distribution. In addition, the Bland–Altman plot was constructed to determine the agreement between the two parameters. Significance was evaluated at the *p* -value less than 0.05 level.

### Ethical Considerations

Ethical approval for the study was obtained from the ethics committee of Fatih Sultan Mehmet Training and Research Hospital (date: 12.03.2015, number: FSM EAH-KAEK/18 SAYI 2015/7). The content and purpose of the study were explained to the participants, and their consent was obtained. The researchers adhered to the tenets of the Declaration of Helsinki throughout the study.

## Results

The study was carried out with a total of 121 cases, 78 (64.5%) male and 43 (35.5%) female, aged 33 to 89 years. The median age of the patients was 67 (25th and 75th percentiles: 60–75) years. Baseline characteristics of the patients are presented in Table 1 .

**Table 1:** Baseline characteristics of the patients

Parameters	
Age (median, 25 ^th^ and 75 ^th^ percentiles)	67 (60–75)
Male ( *n* , %)	78 (64.5%)
Female ( *n* , %)	43 (35.5%)
**Vital parameters** ( **mean/standard deviation)**	
Systolic arterial pressure (mm Hg)	134.13 ± 22.92
Diastolic arterial pressure (mm Hg)	78.55 ± 12.67
Pulse rate (/minute)	102.93 ± 21.56
Respiratory rate (/minute)	27.54 ± 5.98
Body temperature (°C)	36.61 ± 0.59
SpO _2_ levels (%)	85.21 ± 9.5
**Treatments applied in the emergency department (** ***n*** **, %)**	
Intravenous steroid	104 (86 %)
Beta _2_ agonist	120 (99.2 %)
Anticholinergic drug	116 (98.9 %)
Inhaled steroid	35 (28.9 %)
Theophylline	31 (25.6 %)
Noninvasive mechanical ventilation	8 (6.6 %)

Abbreviation: SpO _2_ , oxygen saturation.

Combinations of short-acting inhaled beta _2_ -agonist, anticholinergic, inhaler steroid, and systemic steroid treatments were applied to 64 of the cases (52.9%), and methylxanthine treatment and noninvasive mechanical ventilation were applied to 57 (47.1%) cases in addition to these treatments. Beta-agonists were used in 120 (99.2%) patients, anticholinergics in 116 (98.9%), intravenous steroids in 104 (86%), inhaled steroids in 35 (28.9%), theophylline in 31 (25.6%), and noninvasive mechanical ventilation in 8 (6.6%) patients ( Table 1 ).

**Fig. 1:**
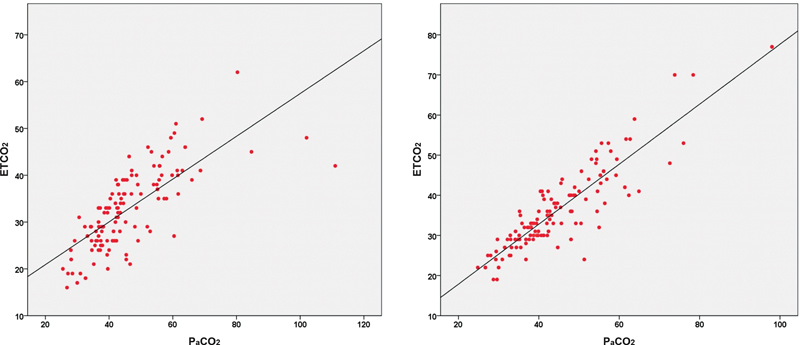
Correlation graphs of partial pressure of carbon dioxide (PaCO _2_ ) and end-tidal carbon dioxide (ETCO _2_ ) before ( **A** ) and after ( **B** ) treatment.

Table 2 presents the comparison of the changes in the pH, PaO _2_ , PaCO _2_ , arterial oxygen saturation (SaO _2_ ), HCO _3_ , and ETCO _2_ values before and after treatment. The correlation between PaCO _2_ and ETCO _2_ was also evaluated. A positive statistically significant correlation was found between the pre-treatment PaCO _2_ and ETCO _2_ values, with the percentage of correlation being determined as 73.6% ( *p* = 0.001; *p* < 0.01, Pearson correlation analysis) ( Fig. 1 ). There was a positive statistically significant correlation between the post-treatment PaCO _2_ and ETCO _2_ values, with a correlation percentage of 88.3% ( *p* = 0.001; *p* < 0.01, Pearson correlation analysis).

**Table 2:** Comparison of changes in the pH, PaO _2_ , PaCO _2_ , SaO _2_ , HCO _3_ , and ETCO _2_ values before and after treatment

	Before treatment (Mean ± SD)	After treatment (Mean ± SD)	*p* -Value
pH	7.4 ± 0.06	7.42 ± 0.05	0.009**
PaO _2_	62.42 ± 18.45	71.31 ± 24.77	0.001**
PaCO _2_	46.04 ± 13.51	44.78 ± 11.96	0.011*
SaO _2_	87.61 ± 9.14	91.52 ± 6.39	0.001**
HCO _3_	29.1 ± 9.73	28.33 ± 5.74	0.248
ETCO _2_	32.79 ± 8.39	36.38 ± 10.12	0.001**

Abbreviations: ETCO _2_ , end-tidal carbon dioxide; HCO _3,_ serum bicarbonate; PaCO _2_ , partial pressure of carbon dioxide; PaO2, partial pressure of oxygen; SaO2, arterial oxygen saturation; SD, standard deviation.

Paired-samples *t* -test; * *p* < 0.05, ** *p* < 0.01.

**Fig. 2.**
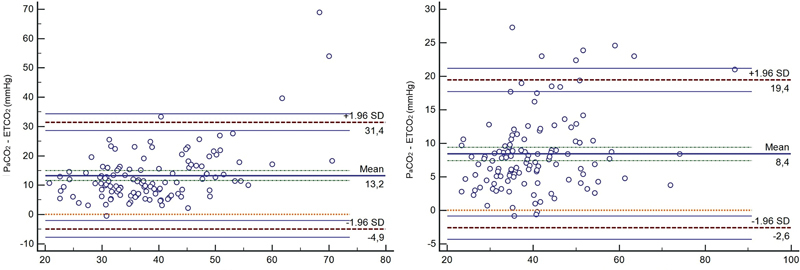
Bland–Altman plot showing the agreement between showing partial pressure of carbon dioxide (PaCO _2_ ) and end-tidal carbon dioxide (ETCO _2_ ) before **(A)** and after **(B)** treatment. SD, standard deviation.

When the Bland–Altman method was used to compare the agreement between the PaCO _2_ and ETCO _2_ parameters before treatment, most of the measurements were found within the limits of agreement (−4.9 and +31.4), and the mean difference was 13.2. The same analysis of the post-treatment values also indicated most measurements were within the limits of agreement (−2.6 and +9.4), with the mean difference being calculated as 8.4. The Bland–Altman plots showing the agreement between the PaCO _2_ and ETCO _2_ parameters before and after treatment are shown in Fig. 2 .

## Discussion

This study investigated whether ETCO _2_ could be used instead of PaCO _2_ , guiding treatment, and determining treatment benefits in patients with COPD exacerbation at the emergency department. We found a statistically significant positive correlation between the PaCO _2_ values obtained by arterial blood gas analysis and ETCO _2_ values obtained by sidestream capnography; nonetheless, we consider that it would not be appropriate to use these values interchangeably in clinical practice.

In a study evaluating 118 patients presenting to the emergency department with COPD exacerbation, Kartal et al found the mean ETCO _2_ value measured by the sidestream method as 33.7 ± 10.45 and the mean PaCO _2_ value as 42.2 ± 13.5. The authors reported that the agreement between ETCO _2_ and PaCO _2_ was low and ETCO _2_ could not be used instead of PaCO _2_ in clinical practice. 8 In a study by Delerme et al, evaluating 48 measurements performed with the sidestream method in 43 patients with shortness of breath and in another study by Jabre et al, in which the authors evaluated the measurements taken by the sidestream method during the ambulance transportation of 49 patients with respiratory distress, the common conclusion was that ETCO _2_ could not clinically replace PaCO _2_ . 9 10 This inconsistency in the measurements made with the sidestream method may be due to various factors, such as air leaks and fluid secretions clogging the measuring tube. Similarly, in both the study of Kartal et al and our study, the difference between the ETCO _2_ and PaCO _2_ values may have arisen due to the cohort consisting of COPD cases. A plausible explanation for this may be the reduced exhalation of CO _2_ in COPD due to both increased dead space and impaired ventilation. During the exacerbation of the disease, CO _2_ excretion is even more limited.

The results of our study were also examined in terms of whether ETCO _2_ values could be used to guide treatment. While the 26 patients with an initial ETCO _2_ value of 40 and above had a mean ETCO _2_ value of 44.5 before treatment, the PaCO _2_ values of the same patients were all above 45, with a mean value of 64.15. Since there was a statistically significant positive correlation between PaCO _2_ and ETCO _2_ , we consider that high ETCO _2_ values can be used in the decision to start noninvasive mechanical ventilation therapy in patients with COPD exacerbation. When the data were examined in terms of the PaCO _2_ values, 49 PaCO _2_ values measured in the pre-treatment period were 45 and above, while the ETCO _2_ values of 23 of the same patients were found to be lower than 40. Therefore, we consider that a low ETCO _2_ value will not be helpful in guiding treatment. In a study conducted by Doğan et al to evaluate the success of ETCO _2_ in showing the severity of COPD exacerbation in 102 patients, it was reported that the ETCO _2_ levels obtained with the mainstream method were higher in those with severe COPD exacerbation. The authors suggested that these high values could be useful in the prehospital setting to determine the need for noninvasive mechanical ventilation or hospitalization, but ETCO _2_ values would provide limited benefits in evaluating patients with COPD exacerbation. 11 In this study, when the Bland–Altman analysis was undertaken to compare the agreement between the PaCO _2_ and ETCO _2_ parameters before treatment, we determined that most measurements were within the limits of agreement (−4.9 and +31.4), and the mean difference was 13.2. Although this is considered to be statistically consistent, due to the mean differences between ETCO _2_ and PaCO _2_ being significantly high, we do not think that the ETCO _2_ value can be used instead of the PaCO _2_ value in the clinical setting.

The percentage of correlation between ETCO _2_ and PaCO _2_ increased after treatment. When the post-treatment agreement between the two parameters was evaluated with the Bland–Altman method, most measurements were found to be within the limits of agreement (−2.6 and +9.4), and the mean difference was 8.4. However, despite the results being statistically significant and the mean difference being reduced compared to the pre-treatment measurements, we consider that ETCO _2_ value cannot be clinically used to replace the PaCO _2_ value because there is still a large mean difference between the two parameters.

In this study, 36 of the 40 patients whose post-treatment ETCO _2_ values were 40 or higher had a PaCO _2_ value of 45 and higher. This suggests that high ETCO _2_ values measured after treatment seem to be effective in deciding whether to continue noninvasive mechanical ventilation therapy. When we evaluated the usability of ETCO _2_ values in the decision to discontinue treatment, we determined that 12 of the 48 patients with a post-treatment PaCO _2_ value of 45 and above had an ETCO _2_ value of below 40. In light of these data, we consider that it is not appropriate to terminate treatment based on lower or decreased ETCO _2_ values compared to the pre-treatment measurements.

There are certain limitations to our study. The first concerns the limited number of patients we were able to reach over the determined study period and the small number of measurements obtained per patient. Due to the observational design of our study, blood gas samples were not taken from the patients without the clinician making this decision, which resulted in a low number of measurement values for comparison. Second, PaCO _2_ values measured in blood gas were not corrected for body temperature. Lastly, we could not classify the cases included in the study according to the severity of COPD exacerbation according to objective data. Therefore, the values of mild and severe cases were evaluated together. On the other hand, our sample with a mean oxygen saturation of 85 and pH of 7.4 seem to be mild COPD exacerbation cases. This may limit the generalizability of the results of our study to patients with severe COPD exacerbations. Studies with subgroup analysis may be better to determine how disease severity affects the relationship between PaCO _2_ and ETCO _2_ .

In conclusion, high ETCO _2_ values may indicate that noninvasive mechanical ventilation should be included in the treatment of patients with COPD without waiting for the results of blood gas analysis. Considering the high hospitalization rate of patients who require noninvasive mechanical ventilation during the exacerbation period, high ETCO _2_ values can be used to predict the need for inpatient treatment. Despite these benefits, ETCO _2_ has limited value in evaluating patients during the COPD exacerbation process and guiding treatment.
